# Supplementary material for: Enhancing predictions of protein stability changes induced by single mutations using MSA-based language models
Source: Bioinformatics. 2024 Jul 16;40(7):btae447. doi: 10.1093/bioinformatics/btae447 (PMC11269464; doi:10.1093/bioinformatics/btae447)
Supplement: btae447_Supplementary_Data [file btae447_supplementary_data.pdf]

Supporting Information

“Enhancing predictions of protein stability changes induced by single mutations using MSA-based Language Models”

Francesca Cuturello [1,*], Marco Celoria [1,2], Alessio Ansuini [1], and Alberto Cazzaniga [1]

[1]AREA Science Park, Località Padriciano 99, 34149 Trieste, Italy
[2]CINECA National Supercomputing Center, Casalecchio di Reno, 40033 Bologna, Italy
[*]Corresponding author. francesca.cuturello@areascienceprk.it

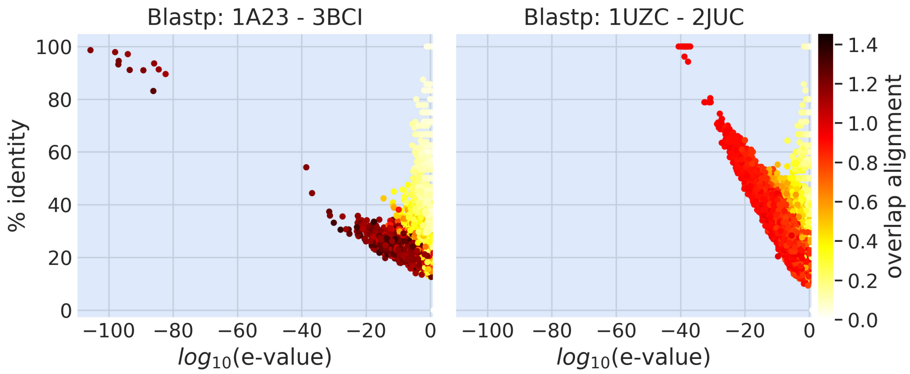

Figure 1: Pairwise Blastp alignment between sequences in MSA of 1A23 (*S1413* training set) vs 3BCI (small test set) (**left**) and 1UZC (*S1413* training set) vs 2JUC (small test set) (**right**).

Table 1: MSAesm_ddG model trained with the symmetrical, reverse and direct $\Delta\Delta G$ distributed *S1413*.

	***S669* test set**		
***S1413* symmetry**	**Pearson r**	**MAE**	**RMSE**
symmetrical	0.50	1.01	1.44
reverse	0.49	1.06	1.47
direct	0.49	1.03	1.47

Table 2: MSAesm_ddG fine-tuned and baseline, trained with different datasets.

	***S669* test set - Ft**			***S669* test set - w/o Ft**		
**Training sets**	**Pearson r**	**MAE**	**RMSE**	**Pearson r**	**MAE**	**RMSE**
S155329	0.53	0.99	1.41	0.53	1.00	1.43
S3648	0.51	0.97	1.41	0.51	0.99	1.41
S1413	0.50	1.01	1.44	0.48	1.03	1.44

Table 3: ESM2_ddG fine-tuned and baseline, trained with different datasets.

	***S669* test set - Ft**			***S669* test set - w/o Ft**		
**Training sets**	**Pearson r**	**MAE**	**RMSE**	**Pearson r**	**MAE**	**RMSE**
S155329	0.49	1.02	1.47	0.51	1.02	1.46
S3648	0.46	1.01	1.46	0.50	1.00	1.43
S1413	0.48	1.03	1.45	0.47	1.03	1.45

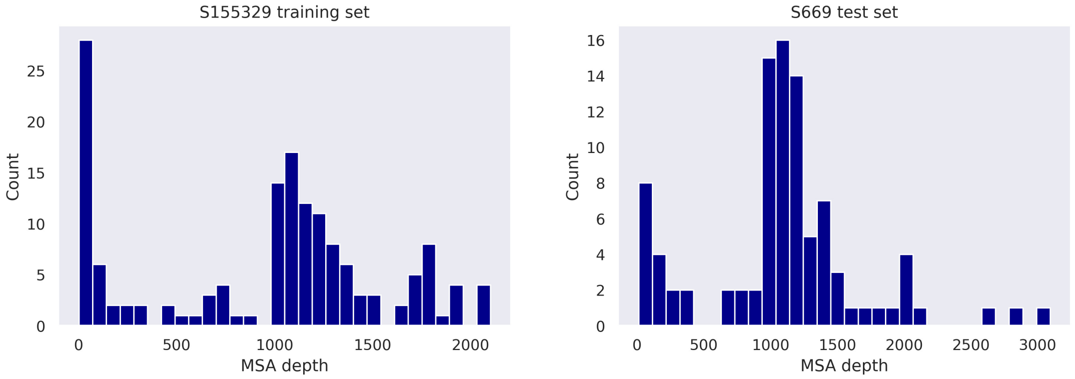

(a) (b)

Figure 2: MSA depth (number of sequences) in the *S155329* training set (2a) and in the *S669* test set (2b).

Tuning Hyperparameters

The Multi-Layer Perceptron block consists of one hidden layer with size 768, followed by a ReLU activation and a dropout layer (with probability p of an element to be zeroed set to $p = 0.2$) that feeds into the final linear layer. We adopt a PyTorch implementation with AdamW optimizer (`torch.optim.AdamW` with default parameters, apart from the learning rate) and OneCycleR learning-rate scheduler (`torch.optim.lr_scheduler.OneCycleLR`). The learning rate is selected for each model and training dataset by means of a grid search with $\texttt{lr} \in \{0.5 \times 10^{-5}, 10^{-5}, 0.5 \times 10^{-4}, 10^{-4}, 0.5 \times 10^{-3}, 10^{-3}, 0.5 \times 10^{-2}, 10^{-2}\}$.
In order to reduce the memory footprint, the PyTorch Automatic Mixed Precision is enabled by means of `torch.autocast` (specifically using `dtype=torch.float16`) in combination with the gradient scaling function `torch.cuda.amp.GradScaler()`. The gradient norm is clipped using `torch.nn.utils.clip_grad_norm_` with `max_norm` $= 10$, to prevent possible exploding gradient problems.
We set a maximum number of 20 epochs for the training in combination with an early stopping criterion. The latter consists in saving the model parameters associated to the epoch with lowest MAE on the validation set, and stopping the training if the MAE on the validation is not decreasing for the next 5 epochs. For training with both the small and designed datasets, we validate on the *ssym* set, which does not overlap with both the training and the test sequences (Figure 3a). When training with the large dataset, a customized validation set is constructed (*S1030*) by excluding from the small dataset sequences that are similar to those in the large dataset (Figure 3b), following the criterion stated in the main text.
Using the PyTorch DistributedDataParallel class (`torch.nn.parallel.DistributedDataParallel`), the fine-tuning is performed on 16 nodes of the LEONARDO Booster partition at CINECA National Supercomputing Center, each consisting of a single socket 32 cores Intel Xeon Platinum 8358 (2.60GHz), together with 4 × NVIDIA A100 GPUs (64GB HBM2e and NVLink 3.0) and 512 GB DDR4 (3200 MHz) RAM. The internal network is provided by NVIDIA Mellanox Infiniband HDR DragonFly+ 200 Gbps.
We consider a batch size $\mathcal{B} = 1$ for a single GPU, so the overall effective batch size $\mathcal{B}_{\text{eff}} = 64$.
Finally, we load the model parameters for the inference on the test sets on a single GPU.

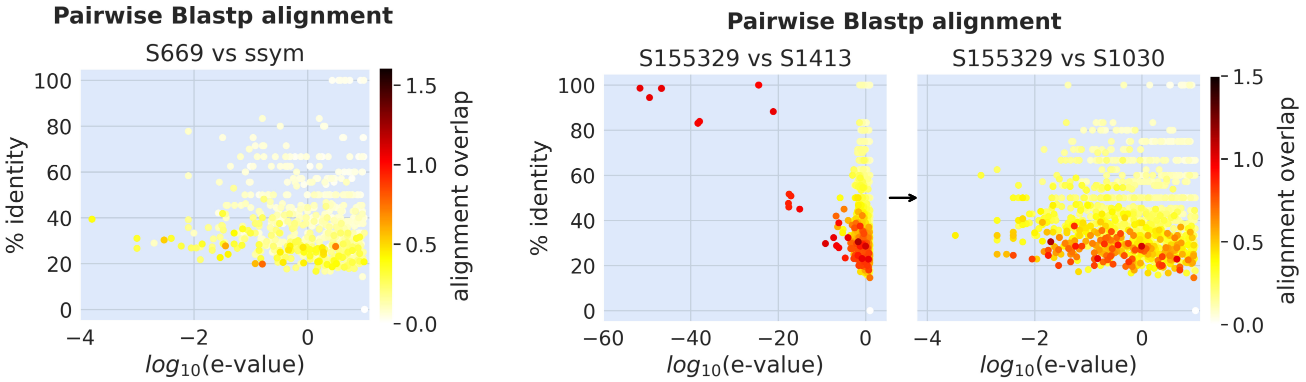

(a) (b)

Figure 3: Pairwise BLASTp alignments (% identity, e-value and alignments overlap). 3a: *S669* test set against the ssym set. 3b: *S155329* training set against the *S1413* dataset, before (**left**) and after (**right**) filtering by similarity to obtain the *S1030* dataset (used for validation in the *S155329* training).

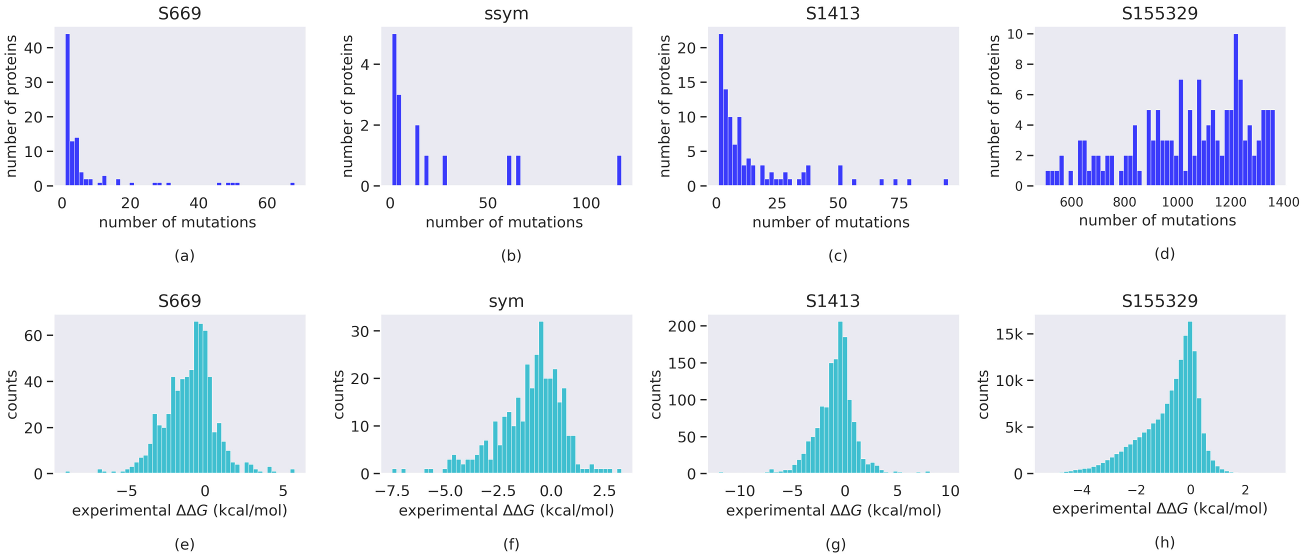

Figure 4: Number of proteins vs number of mutations (a-d) and $\Delta\Delta G$ distribution (e-h) for all datasets.
